# Supplementary material for: Structural characterization and AlphaFold modeling of human T cell receptor recognition of NRAS cancer neoantigens
Source: Sci Adv. 2024 Nov 22;10(47):eadq6150. doi: 10.1126/sciadv.adq6150 (PMC11584006; doi:10.1126/sciadv.adq6150)
Supplement: Supplementary file 1 — Figs. S1 to S6 Tables S1 to S11 [file sciadv.adq6150_sm.pdf]

Science Advances
AAAS

Supplementary Materials for

Structural characterization and AlphaFold modeling of human T cell receptor recognition of NRAS cancer neoantigens

Daichao Wu *et al.*

Corresponding author: Daichao Wu, wudaichao@usc.edu.cn; Roy A. Mariuzza, rmariuzz@umd.edu; Brian G. Pierce, pierce@umd.edu

Sci. Adv. **10**, eadq6150 (2024)
DOI: 10.1126/sciadv.adq6150

This PDF file includes:

Figs. S1 to S6
Tables S1 to S11

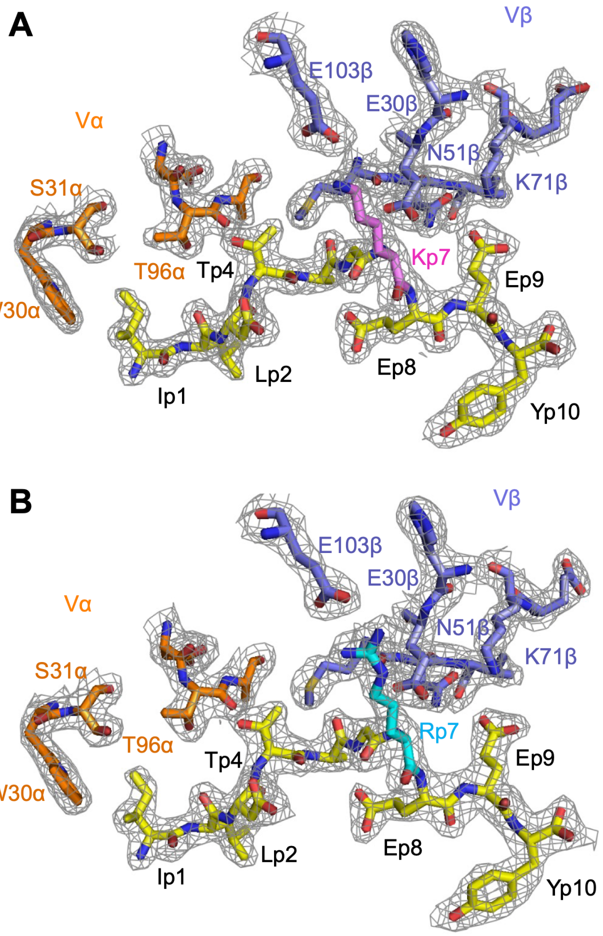

Fig. S1. Electron density in the interfaces of the N17.1.2–NRAS^{Q61K}–HLA-A1 and N17.1.2–NRAS^{Q61R}–HLA-A1 complexes. (A) Electron density in the interface of the N17.1.2–NRAS^{Q61K}–HLA-A1 complex. Density from the final $2F_o - F_c$ map at 2.10 Å resolution is contoured at 1σ. **(B)** Electron density in the interface of the N17.1.2–NRAS^{Q61R}–HLA-A1 complex. Density from the final $2F_o - F_c$ map at 2.26 Å resolution is contoured at 1σ. Carbon atoms are brown (N17.1.2 α chain), blue (N17.1.2 β chain), yellow (NRAS peptide); violet (Q61K mutant residue), or cyan (Q61R mutant residue).

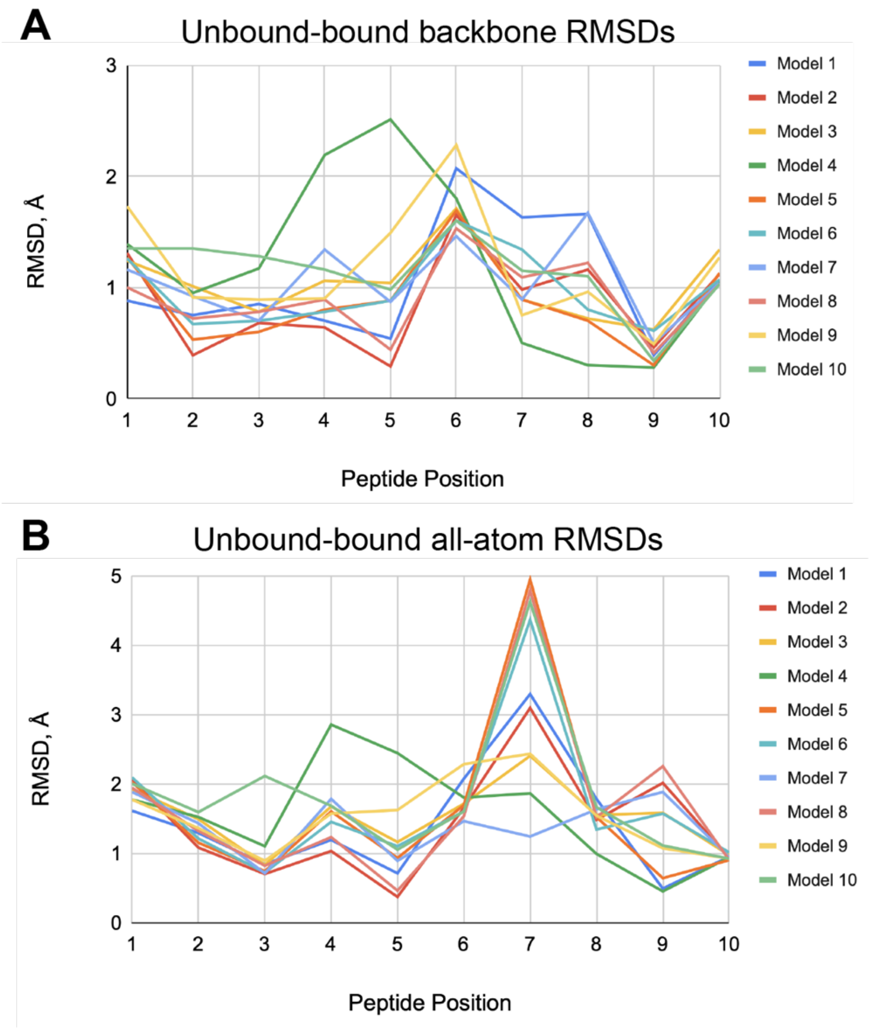

Fig. S2. NRAS^{Q61K} peptide per-residue root-mean-square distances (RMSDs) between N17.1.2 TCR-bound structure and unbound structure NMR models. (A) Backbone atom and **(B)** all-atom RMSD values were calculated for each peptide position after superposition of MHC in the N17.1.2–NRAS^{Q61K}–HLA-A1 complex and MHC of unbound NRAS^{Q61K}–HLA-A1 (PDB accession code 6MPP) (*20*). Per-residue RMSDs were calculated between TCR-bound peptide and each of 10 NMR models of the unbound NRAS^{Q61K}–HLA-A1 structure (lines colored separately, as shown on right).

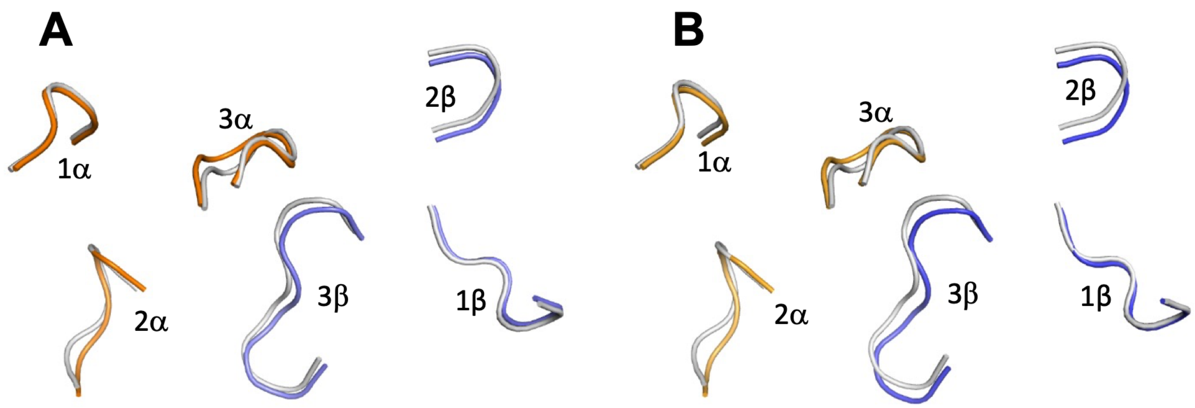

Fig. S3. Comparison of CDR loops in unbound versus bound TCR N17.1.2. (A) Superposition of CDR loops of unbound N17.1.2 (gray) onto CDR loops of N17.1.2 bound to $NRAS^{Q61K}$–HLA-A1 (Vα CDRs, orange; Vβ CDRs, blue). **(B)** Superposition of CDR loops of unbound N17.1.2 (gray) onto CDR loops of N17.1.2 bound to $NRAS^{Q61R}$–HLA-A1 (Vα CDRs, orange; Vβ CDRs, blue).

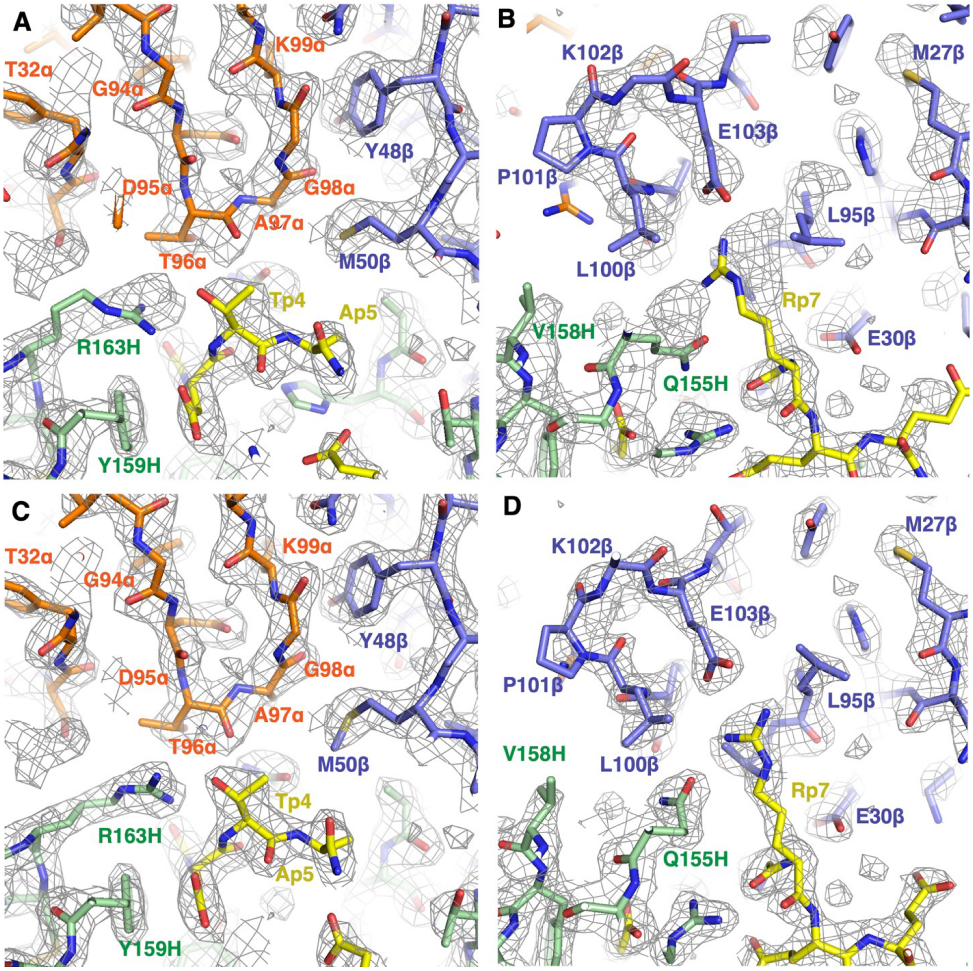

Fig. S4. Comparison of AlphaFold prediction of TCR N17.1.2–NRAS^{Q61R}–HLA-A1 complex generated by TCRmodel2 with crystallographic density map and structures. (A) AlphaFold prediction of N17.1.2–NRAS^{Q61R}–HLA-A1 complex compared with experimental electron density map in the region of CDR3α (Vα, orange; Vβ, blue; NRAS^{Q61R} peptide, yellow; HLA-A1, green). **(B)** AlphaFold prediction of N17.1.2–NRAS^{Q61R}–HLA-A1 complex compared with electron density map in the region of CDR3β. **(C)** Model of N17.1.2–NRAS^{Q61R}–HLA-A1 complex built into electron density map in the region of CDR3α (Vα, orange; Vβ, blue; NRAS^{Q61R} peptide, yellow; HLA-A1, green). **(D)** Model of N17.1.2–NRAS^{Q61R}–HLA-A1 complex built into electron density map in the region of CDR3β.

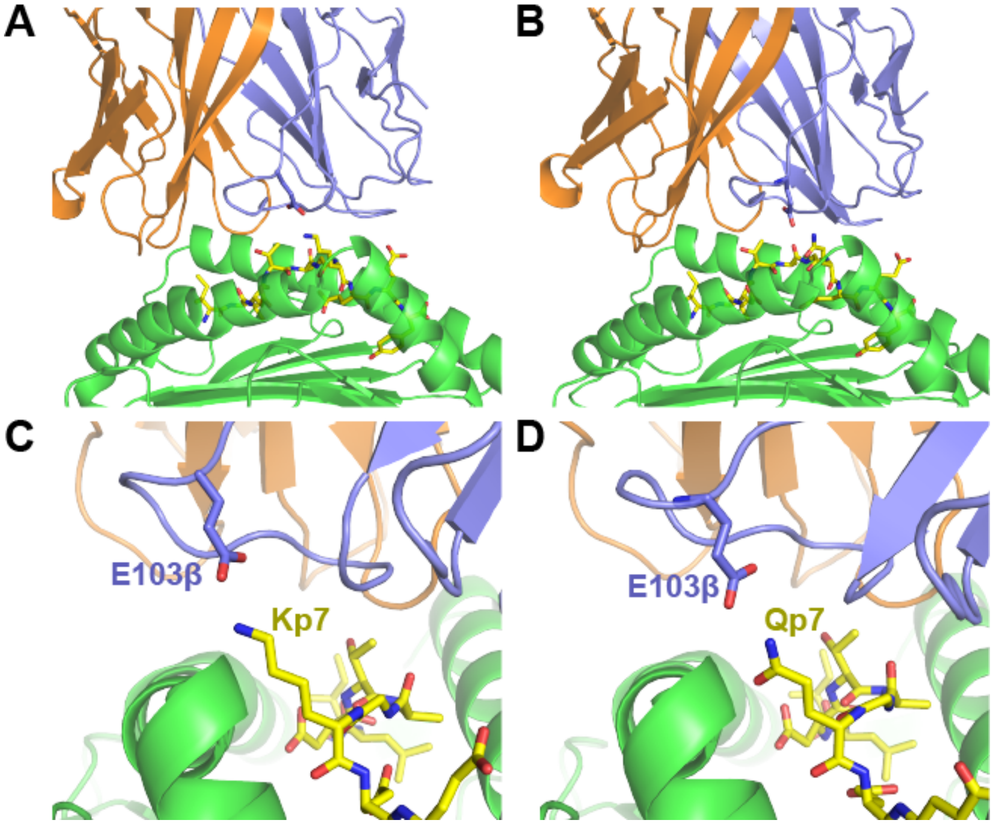

Fig S5. Model of N17.1.2-NRAS^{Q61wt}-HLA-A1 complex. (A) The X-ray structure of the N17.1.2-NRAS^{Q61K} -HLA-A1 X-ray structure is shown in comparison with (B) a model of the N17.1.2-NRAS^{Q61wt}-HLA-A1 complex from TCRmodel2. A close-up view of the interface of the (C) Q61K complex and (D) wild-type complex interfaces is also shown, with TCRβ E103 and peptide K7/Q7 residues labeled. TCRs are orange (α chain) and slate (β chain), peptides are yellow, and HLA-A1 MHCs are green. AlphaFold2 scores for the modeled complex are 0.91 (model confidence) and 91.4 (I-pLDDT), and the NRAS^{Q61wt} complex model has 0.81 Å interface RMSD with the NRAS^{Q61K} X-ray complex.

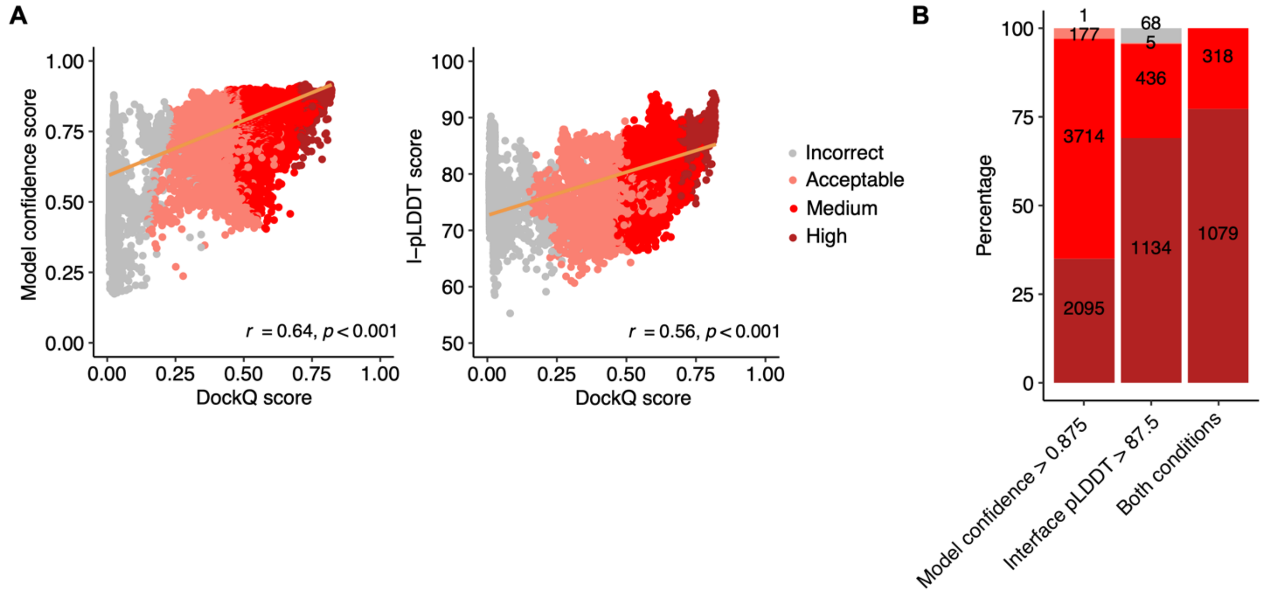

Fig S6. Score versus accuracy for TCR-pMHC benchmark models. (A) 1000 TCRmodel2 models were generated for each of 20 benchmark complexes (from (*25*)), and AlphaFold2 model confidence scores (left) and interface pLDDT (I-pLDDT) scores (right) are shown compared with model accuracy (DockQ score) for each model. Points are colored by CAPRI accuracy levels, as indicated on the right. Best fit regression lines (orange) and Pearson correlation values, and correlation p-values shown for reference. (B) Accuracy rates for pooled benchmark models with the specified AlphaFold score cutoffs for model confidence, interface pLDDT (I-pLDDT), or combination of both individual cutoffs. The stacked bars denote the proportion of Incorrect, Acceptable, Medium, and High CAPRI accuracy criteria predictions for the cutoff. Numerical labels indicate the model counts for each accuracy level.

	N17.1.2–Q61K–HLA-A1	N17.1.2–Q61R–HLA-A1	N17.1.2
PDB	8YIV	8YJ2	8YJ3
**Data collection**			
Resolution range (Å)	42.7–2.10 (2.18–2.10)	42.8–2.26 (2.34–2.26)	47.6–3.50 (3.63–3.50)
Space group	*C* 2 2 21	*C* 2 2 21	*P* 1 21 1
Unit cell parameters	71.1, 133.2, 222.5<br>90, 90, 90	71.5, 134.2, 222.1<br>90, 90, 90	103.2, 66.1, 138.6,<br>90, 97.8, 90
Total reflections[a]	387,710 (25,546)	464,104 (24,741)	148,044 (14,845)
Unique reflections[a]	61,763 (6,011)	49,624 (4,524)	23,156 (2,248)
Multiplicity[a]	6.3 (4.2)	9.3 (5.5)	6.4 (6.6)
Completeness (%)[a]	99.8 (99.0)	98.5 (91.0)	97.7 (97.3)
Mean $I/\sigma(I)$[a]	13.7 (2.2)	13.5 (2.2)	9.8 (1.7)
Wilson *B* factor ($Å^2$)	33.9	32.4	112.0
R_{merge}[a,b]	0.080 (0.403)	0.124 (0.490)	0.206 (0.821)
CC1/2	0.992 (0.857)	0.985 (0.883)	0.953 (0.777)
**Refinement**			
R_{work}[c]	0.191 (0.228)	0.189 (0.228)	0.258 (0.334)
R_{free}[c]	0.226 (0.305)	0.229 (0.263)	0.316 (0.366)
No. of protein atoms	6,683	6,680	14,248
No. of waters	547	426	0
Protein residues	835	832	1796
r.m.s.d. from ideality			
Bond lengths (Å)	0.011	0.009	0.004
Bond angles (°)	1.46	1.31	1.10
Ramachandran plot statistics			
Favored (%)	97.0	97.0	95.0
Allowed (%)	3.0	3.0	5.0
Disallowed (%)	0	0	0
Clashscore	8.8	5.6	6.8
Average *B* factor ($Å^2$)	39.4	35.8	125.0
Protein	39.1	35.7	125.0
Waters	41.7	37.8	–

Table S1. Data collection and refinement statistics

[a]Values in parentheses correspond to the highest resolution shell.
[b]$R_{merge} = \sum|I_j - \langle I \rangle|/\sum I_j$, where I_j is the intensity of an individual reflection and $\langle I \rangle$ is the average intensity of that reflection.
[c]R_{work} (R_{free}) $= \sum||F_o| - |F_c||/\sum|F_o|$; 5.0% of data were used for R_{free}.

Complex PDB[1]	TCR position, Å[2]	% Peptide Contacts[3]
4JRY	-14.6	20
6TRO	-6.1	35
**N17.1.2–Q61R–HLA-A1**	**-3.6**	26
6ULR	-3.5	17
**N17.1.2–Q61K–HLA-A1**	**-2.8**	22
3FFC	-2.3	29
6AVF	-1.7	36
4QRP	-0.9	36
2NX5	-0.7	38
8GVB	-0.5	19
1AO7	-0.4	39
3HG1	-0.2	42
5BRZ	0.1	14
6R2L	0.4	32
3UTS	0.4	59
1KJ2	0.7	19
2AK4	1.2	68
5WKH	1.2	32
3QDG	1.3	40
3VXR	1.5	42
3O4L	1.7	31
3VXU	1.9	24
6AVG	2.0	40
5NHT	2.7	46
3VXM	2.7	60
7PHR	2.8	24
7L1D	2.9	27
3MV7	3.1	62
5XOV	3.6	45
6RSY	3.6	36
6BJ3	3.8	30
4MNQ	3.9	45
6VMX	4.0	37
1LP9	4.0	34
7RRG	4.1	11
8DNT	4.1	40
6RP9	4.2	56
6VM8	4.3	48
5JHD	4.5	27
5ISZ	4.6	36
3QDM	4.9	51
7DZM	5.2	46
6P64	5.2	50
5JZI	5.2	34
5WLG	5.3	27
4MJI	5.4	27
7N6E	5.4	52
2YPL	5.5	52

5EU6	5.8	40
1OGA	5.8	35
6MTM	5.8	25
4EUP	5.8	56
7NDQ	5.9	23
2BNQ	5.9	56
1G6R	6.0	49
3SJV	6.2	29
7N1F	6.2	58
7PDW	6.3	48
2ESV	6.3	45
6RPB	6.4	40
5TJE	6.4	40
7N2N	6.9	36
8D5Q	7.0	56
5D2N	7.1	34
3GSN	7.6	47
6RPA	7.7	40
7OW5	7.8	26
1FO0	9.6	42
7RK7	9.6	38
6ZKZ	9.7	23
6VRM	9.8	41
5W1W	10.4	30
1MI5	10.6	17
6L9L	10.6	62
7N4K	10.7	62
4QRR	10.9	33
5IVX	10.9	44
7NA5	11.9	47
7N1E	12.0	59
4G8G	12.2	29
3TFK	12.4	33
5TEZ	13.0	38
6VRN	14.8	68
5SWS	22.3	25

Table S2. MHC peptide groove axis position, and percentage of peptide contacts, of TCR in N17.1.2 complex structures and 82 reference TCR–peptide–MHC class I complex structures

[1]Protein Data Bank (PDB) code of TCR–pMHC complex structure, or structure from this study (N17.1.2–Q61K–HLA-A1, N17.1.2–Q61R–HLA-A1; bold). Complexes from PDB are a set of 82 nonredundant TCR–pMHC class I structures from TCR3d (*32*).
[2]Position on MHC peptide groove axis, in Ångstroms, of TCR variable domain center projected onto MHC plane, as previously described (3), based on the reference frames in the TCR3d database (4). The axis is oriented with peptide C-terminus in the positive direction. Structures are listed in order of position.
[3]Percent of peptide contacts among total TCR atomic contacts (< 4 Å) to peptide and MHC.

HLA-A1	N17.1.2–Q61K		N17.1.2–Q61R	
	Hydrogen bonds	Van der Waals contacts	Hydrogen bonds	Van der Waals contacts
α1				
E55H	W30α(Nε1) E55H(Oε2)	W30α(3)	W30α(Nε1) E55H(Oε2)	W30α(3)
E58H	S28α(Oγ) E58H(Oε2),<br>S28α(Oγ) E58H(Oε1),<br>W29α(Nε1) E58H(Oε1)	S28α(2),<br>W29α(3)	S28α(Oγ) E58H(Oε2),<br>S28α(Oγ) E58H(Oε1)	S28α(2),<br>W29α(1)
Y59H		W30α(3)		W30α(3)
Q62H	T96α(N) Q62H(Oε1),<br>T96α(Oγ1) Q62H(Oε1),<br>T96α(Oγ1) Q62H(Nε2)	D95α(2),<br>T96α(6)	T96α(N) Q62H(Oε1),<br>T96α(Oγ1) Q62H(Oε1),<br>T96α(Oγ1) Q62H(Nε2)	D95α(3),<br>T96α(6),<br>A97α(1)
R65H	Y48β(Oη) R65H(Nη1),<br>D95α(Oδ2) R65H(Nη1),<br>D95α(Oδ2) R65H(Nη2),	Y48β(3),<br>D56β(6),<br>D95α(3),<br>A97α(3)	Y48β(Oη) R65H(Nη1),<br>D95α(Oδ2) R65H(Nη1),<br>D95α(Oδ2) R65H(Nη2),<br>A97α(O) R65H(Nη1)	Y48β(3),<br>D56β(6),<br>D95α(3),<br>A97α(2),<br>K99α(1)
N66H	T96α(O) N66H(Nδ2)	M50β(1),<br>T96α(3),<br>A97α(3)	T96α(O) N66H(Nδ2)	M50β(1),<br>T96α(4),<br>A97α(3)
K68H	T55β(O) K68H(Nζ),<br>D56β(Oδ1) K68H(Nζ),<br>D56β(Oδ2) K68H(Nζ)	D56β(2),	T55β(O) K68H(Nζ),<br>D56β(Oδ1) K68H(Nζ),<br>D56β(Oδ2) K68H(Nζ)	D56β(2),
A69H		M50β(1)		M50β(1)
Q72H		V52β(1),<br>E53β(1),<br>V54β(2)		V52β(1),<br>E53β(3),<br>V54β(2)
T73H				N51β(1)
α2				
E154H	K102β(Nζ) E154H(Oε1),<br>K102β(Nζ) E154H(Oε2)	L100β(3),<br>K102β(2)	K102β(Nζ) E154H(Oε2)	L100β(2),<br>K102β(3)
Q155H	T98β(O) Q155H(Nε2),	V96β(3),<br>T98β(1),<br>L100β(5),	T98β(O) Q155H(Nε2),	V96β(2),<br>T98β(1),<br>L100β(2)
V158H				L100β(1)
G162H		D54α(2)		
R163H	Y33α(Oη) R163H(Nη2)	S31α(2),<br>D54α(2),<br>T96α(7)		S31α(1),<br>D54α(1),<br>T96α(8),<br>T98β(1)
D166H	D54α(Oδ1) D166H(Oδ1),<br>D54α(Oδ1) D166H(Oδ2)	W30α(1),<br>D54α(5)	D54α(Oδ1) D166H(Oδ2)	W30α(2),<br>D54α(9)
G167H		W30α(2)		W30α(2)
R170H	W30α(Nε1) R170H(Nε)	W30α(22)	W30α(Nε1) R170H(Nε)	W30α(23)
Y171H		W30α(2)		W30α(2)

Table S3. Interactions between N17.1.2 and HLA-A1

Contact residues were identified with the CONTACT program (*48*). Hydrogen bonds were calculated using a cut-off distance of 3.5 Å. The cut-off distance for van der Waals contacts was 4 Å.

Mutant[1]	N17.1.2–NRAS^{Q61K}–HLA-A1 ΔΔG[2]	N17.1.2–NRAS^{Q61R}–HLA-A1 ΔΔG[2]
E55A	1.1	1.2
E58A	1.0	1.2
Y59A	0.5	0.5
Q62A	1.1	1.1
E63A	0.2	0.1
R65A	2.4	2.4
N66A	0.6	0.1
K68A	0.9	1
A69G	0.6	0.6
Q72A	0.7	1.1
T73A	0	0
V150A	0.1	0
H151A	0	0
E154A	0.1	0.1
Q155A	1.6	0.8
V158A	0.1	0.3
G162A	-0.1	0.1
R163A	2.1	2.0
D166A	0.3	0
G167A	-0.2	-0.2
R170A	1.1	1.5
Y171A	0.5	0.4

Table S4. TCR binding affinity changes from computational alanine scanning of HLA-A1 MHC residues

[1]MHC residue alanine substitutions for all MHC residues proximal to the TCR (< 5 Å) in the N17.1.2–NRAS^{Q61K}–HLA-A1 complex structure. Wild-type alanine residues were mutated to glycine.

[2]Alanine (or glycine, for wild-type alanine residues) substitutions were modeled using a protocol in Rosetta (*33, 53*) to compute the TCR binding energy change (ΔΔG). Values are in Rosetta Energy Units (REU), comparable to kcal/mol. Predicted hotspots (ΔΔG ≥ 0.8 REU) are highlighted in red.

Q61K/R	N17.1.2–Q61K		N17.1.2–Q61R	
	Hydrogen bonds	Van der Waals contacts	Hydrogen bonds	Van der Waals contacts
I1p		W30α(3), S31α(1)		W30α(3), S31α(2)
T4p	T96α(O) T4p(Oγ1)	T96α(4) T98β(4)	T96α(O) T4p(Oγ1)	T96α(4) T98β(5)
G6p		E30β(3), V96β(1)		E30β(4)
K/R7p	E30β(Oε1) K7p(N), E30β(Oε2) K7p(N), E103β(Oε1) K7p(Nζ), E103β(Oε2) K7p(Nζ)	E30β(2), L95β(1), V96β(2), L100β(2), E103β(2)	E30β(Oε2) R7p(N), E103β(Oε1) R7p(Nη1), E103β(Oε2) R7p(Nη1), E103β(Oε1) R7p(Nη2)	E30β(3), L95β(2), V96β(5), L100β(2), E103β(5)
E9p	N51β(Oδ1) E9p(Oε2), N51β(Nδ2) E9p(Oε2), K71β(Nζ) E9p(Oε2)	N51β(1), K71β(3)	N51β(Nδ2) E9p(Oε1), K71β(Nζ) E9p(Oε1)	N51β(2), K71β(2)

Table S5. Interactions between TCR N17.1.2 and NRAS$^{Q61K/R}$ peptide

Contact residues were identified with CONTACT (*48*). Hydrogen bonds were calculated using a cut-off distance of 3.5 Å. The cut-off distance for van der Waals contacts was 4 Å.

Mutant	N17.1.2–NRAS^{Q61K}–HLA-A1[1]	N17.1.2–NRAS^{Q61R}–HLA-A1[1]
I55A	0.8	0.7
L56A	0	0
D57A	0.2	0.3
T58A	2.1	2.0
A59G	0.1	0.1
G60A	-0.2	-0.3
K61A/R61A	1.4	1.8
E62A	0.2	0.3
E63A	0.9	0.9
Y64A	0	0
K61Q/R61Q	1.2	1.7

Table S6. TCR binding affinity changes from computational alanine and reversion (K61Q/R61Q) mutagenesis of peptide

[1]Alanine (or glycine, for wild-type alanine residues) substitutions, or wild-type glutamine reversion substitutions (K61Q, R61Q), were modeled using a protocol in Rosetta (*33, 53*) to compute the TCR binding energy change ($\Delta\Delta G$). Values are in Rosetta Energy Units (REU), comparable to kcal/mol. Predicted hotspots ($\Delta\Delta G \geq 0.8$ REU) are highlighted in red.

N17.1.2–NRAS^{Q61K}–HLA-A1

**Peptide residue**	**X-ray**	**TCRmodel2**
T4p	**T96α(O) T4p(Oγ1)**	**T96α(O) T4p(Oγ1)**
K7p	E30β(Oε1) K7p(N)	
	**E30β(Oε2) K7p(N)**	**E30β(Oε2) K7p(N)**
	**E103β(Oε1) K7p(Nζ)**	**E103β(Oε1) K7p(Nζ)**
	E103β(Oε2) K7p(Nζ)	
E9p	N51β(Oδ1) E9p(Oε2)	
	N51β(Nδ2) E9p(Oε2)	
	**K71β(Nζ) E9p(Oε2)**	**K71β(Nζ) E9p(Oε2)**
		K71β(Nζ) E9p(Oε1)
		N28β(Nζ) E9p(Oε1)

N17.1.2–NRAS^{Q61R}–HLA-A1

**Peptide residue**	**X-ray**	**TCRmodel2**
T4p	**T96α(O) T4p(Oγ1)**	**T96α(O) p4T(Oγ1)**
R7p	**E30β(Oε2) R7p(N)**	**E30β(Oε2) R7p(N)**
		E30β(Oε1) R7p(N)
	E103β(Oε1) R7p(Nη1)	
	E103β(Oε2) R7p(Nη1)	
	**E103β(Oε1) R7p(Nη2)**	**E103β(Oε1) R7p(Nη2)**
		E103β(Oε2) R7p(Nη2)
E9p	N51β(Nδ2) E9p(Oε1)	
	**K71β(Nζ) E9p(Oε1)**	**K71β(Nζ) E9p(Oε1)**
		K71β(Nζ) E9p(Oε2)

Table S7. TCR–peptide polar and charged atomic contacts in X-ray and AlphaFold (TCRmodel2) modeled interfaces

Contacts were identified using the CONTACT program (*48*). Matching contacts between modeled and X-ray structures are shown on same line and in bold.

TCR name	α chain Vα	CDR3α	Jα	β chain Vβ	CDR3β	Jβ
N17.1.2	TRDV1	CALGDTAGKSTF	TRAJ27-1	TRBV27	CASSLVSTPLPKETQYF	TRBJ2-5
N17.2	TRAV5	CAESSGGGFKTIF	TRAJ9	TRBV6-6	CASSTPGPSAYEQYF	TRBJ2-7
N17.3.2	TRAV19	CALSESGDAAGNKLTF	TRAJ17	TRBV6-1	CAEGENTEAFF	TRBJ1-1
N17.5	TRAV19	CALSETINNAGNMLTF	TRAJ39	TRBV6-6	CASSQNTEAFF	TRBJ1-1
N135.1	TRAV19	CALSESHNNAGNMLTF	TRAJ39	TRBV6-1	CAASQNTEAFF	TRBJ1-1

Table S8. Sequence information for TCRs that target NRAS^{Q61K}–HLA-A1, from Peri et al. (*15*)

Case	Model Accuracy[1]				Models Above Cutoff[2]	
	Incorrect	Acceptable	Medium	High	All	High
7rrg	156	178	666	0	0	0
7sg0	0	2	998	0	39	0
7ndq	2	190	808	0	2	0
6zkw	6	434	444	116	5	4
7na5	696	262	34	8	0	0
7dzm	409	256	305	30	8	8
7qpj	8	356	395	241	176	121
7ow5	347	11	483	159	0	0
8gom	243	258	499	0	0	0
7sg1	1	413	586	0	117	0
7rk7	0	1000	0	0	0	0
7phr	84	0	0	916	511	511
7n2n	498	483	19	0	0	0
7t2b	1	189	810	0	0	0
8gvb	1	17	982	0	0	0
7rdv	0	0	582	418	189	144
7t2c	3	0	997	0	0	0
7z50	0	0	279	721	350	291
7l1d	77	438	485	0	0	0
8d5q	1	682	317	0	0	0

Table S9. Accuracy levels of TCRmodel2 benchmark case models for total and filtered sets.

[1]CAPRI accuracy levels are shown for the 1000 TCRmodel2 models for each TCR-pMHC benchmark complex.
[2]Number of total models and number of high accuracy models for each case above the proposed score cutoff (model confidence > 0.875 and I-pLDDT > 87.5).

Score	N17.1.2-Q61K-HLA-A1 Top Model[1]	N17.1.2-Q61K-HLA-A1 # High Top 5[2]	N17.1.2-Q61R-HLA-A1 Top Model[1]	N17.1.2-Q61R-HLA-A1 # High Top 5[2]
Model confidence	High	4	High	5
I-pLDDT	High	4	High	5
Rosetta REF15 (*40*)	Medium	3	Medium	0
ZRANK2 (*41*)	Acceptable	1	Acceptable	0

Table S10. Ranking accuracy for N17.1.2 TCR-pMHC complex models.

[1]TCRmodel2 models (1000 per complex) were ranked by the given score, and CAPRI accuracy of the top-ranked model is shown.
[2]Number of high accuracy models in the top 5 ranked models. The total numbers of high accuracy models are 10 (N17.1.2-Q61K-HLA-A1) and 7 (N17.1.2-Q61R-HLA-A1) for each set of 1000 models.

	TCRmodel2		AlphaFold2.3		AlphaFold3	
# Preds[1]	1000		200		5	
Complex[2]	Model conf[3]	I-pLDDT[3]	Model conf[3]	I-pLDDT[3]	Model conf[3]	I-pLDDT[3]
N17.1.2	0.917	93.5	0.887	81.87	0.94	89.05
N17.2	0.866	85.29	0.832	76.03	0.74	67.62
N17.3.2	0.885	82.88	0.856	79.59	0.83	71.49
N17.5	0.833	74.77	0.863	82.56	0.88	77.52
N135.1	0.863	83.11	0.815	77.68	0.87	75.77

Table S11. Confidence scores of modeled TCR–NRAS^{Q61K}–HLA-A1 complexes

[1]Number of models generated per complex for the protocol.
[2]Complex being modeled, denoted by TCR name. All TCRs modeled in complex with NRAS^{Q61K}–HLA-A1 target. TCR N17.1.2 scores shown for comparison, while predictive modeling was performed for other NRAS^{Q61K}–HLA-A1 TCRs described in Peri et al. (*15*).
[3]AlphaFold model confidence and interface pLDDT (I-pLDDT) scores are shown for the top-ranked model generated for the complex with the given protocol. For AlphaFold3, the provided "ranking_score", which has a slight modification of the AlphaFold2.3 model confidence score calculation, is shown for model confidence. Cells highlighted in green in cases with both score criteria were met (model confidence > 0.875, I-pLDDT > 87.5).
